# The Mediating Effect of Perceived Social Support on Mental Health and Life Satisfaction among Residents: A Cross-Sectional Analysis of 8500 Subjects in Taian City, China

**DOI:** 10.3390/ijerph192214756

**Published:** 2022-11-10

**Authors:** Yaru Dong, Lingzhong Xu, Shoucai Wu, Wenzhe Qin, Fangfang Hu, Menghua Li, Yanrui Xu

**Affiliations:** 1 Centre for Health Management and Policy Research, School of Public Health, Cheeloo College of Medicine, Shandong University, Jinan 250012, China; 2 National Health Commission (NHC) Key Lab of Health Economics and Policy Research, Shandong University, Jinan 250012, China; 3 Department of Geriatrics, Qilu Hospital, Cheeloo College of Medicine, Shandong University, Jinan 250012, China

**Keywords:** perceived social support, mental health, life satisfaction, residents, cross-sectional study

## Abstract

Several studies have explored the relationship between mental health and life satisfaction. However, few studies have clarified the mechanisms underlying the relationship between mental health and life satisfaction among a large sample of the whole population. The aim of this study was to explore the mediating role of perceived social support between mental health and life satisfaction among the residents in Taian City, China. A total of 8500 residents were included in the analysis. A descriptive analysis was conducted to describe the sample characteristics. Pearson correlation was employed to explore the correlation between mental health and life satisfaction. The mediating role of perceived social support was analyzed using SPSS26.0. This study found that the residents’ average score of life satisfaction was 24.60 ± 4.12. Mental health was significantly correlated with perceived social support and life satisfaction. After adjusting for controlling variables, perceived social support played a partially mediating effect on mental health and life satisfaction, accounting for 21.04% of the total effect. However, data are cross-sectional, and causal conclusions cannot be drawn. Attention should be paid to the residents’ mental health and intervention should be considered for residents with mental disorders to improve the residents’ life satisfaction.

## 1. Introduction

As a result of the rapid development of science and technology and the accelerating pace of life, people are facing an increasing number of mental health problems. Furthermore, changes in society have triggered new thinking about life and what it means for everyone. Therefore, residents’ mental health and life satisfaction have been the focus of academic research and attention from all walks of life.

The World Health Organization (WHO) recognized the essential role of mental health for achieving overall health, and mental health has been incorporated into population health strategies by various jurisdictions [1]. A landmark paper by Prince and colleagues surmised that there was “no health without mental health” [2]. Mental health has received increasing attention in recent years. It is moving up the policy agenda and is increasingly recognized as a key public health indicator [3]. Mental health status is determined by the cumulative effect of risk and protective factors that affect an individual’s ability to adapt to new situations and develop healthy coping mechanisms. Mental disorders are projected to become the most common medical condition by 2030 [3]. Studies showed that one in eight people, or 970 million people worldwide, had a mental disorder [4].

Life satisfaction is a crucial facet of subjective well-being [5]. The annual World Happiness Report provides self-reported life satisfaction scores of more than 150 countries to reflect social progress [6]. Population-level life satisfaction scores are widely reported, and studies support their validity [7]. Life satisfaction refers to a cognitive and global evaluation of the quality of one’s life as a whole [8]. It also serves as an indicator of one’s life quality and an important parameter to measure people’s life quality. More specifically, people’s life satisfaction is influenced by many factors, such as psychiatric symptoms, social self-efficacy, social support, and community integration [9,10]. Research on the determinants of life satisfaction shows that health status is one of the most important factors associated with life satisfaction [11]. Moreover, a strong and robust inverse association exists between life satisfaction and the presence of mental illness [12,13,14]. Furthermore, life satisfaction is related to social engagement [15,16]. People tend to gain more opportunities through social experiences within the community, which ultimately leads to higher life satisfaction [17].

Perceived social support is viewed as “the perception of an individual about the amount and quality of support received from their social network”, while received social support is defined as “the objective quantification of the help and aid received from their social network” [18]. Perceived social support is the most commonly measured index of social support [19]. It can help individuals moderate perceived stress [20]. The relationship between perceived social support and mental health has been extensively studied [21,22,23]. Social support may have a positive effect on enhancing mental health when an individual experiences high stress [24].

Numerous studies have explored the relationship among mental health, social support, and life satisfaction. Chappell, N. L examined the predictors of subjective quality of life and life satisfaction. It was found that social support and health predicted life satisfaction in China and Canada [25]. Kant, S explored the contributions of social, cultural, and land use to aboriginal well-being and health in Canada. Results revealed that reducing the prevalence of psychological problems by investing in the improvement of health services contributed to satisfaction [26]. Wei-Wei, D explored socio-cultural adaptation and the overall self-perceived quality of life by older Chinese women and men emigrating from mainland China to Canada. His study showed that physical and mental health, family relationships, and social support or community relationships affected their life satisfaction [27]. De Leo studied 586 individuals in Italy, the Netherlands, and Finland, and results confirmed that good health had a significant impact on the quality of life of older people [28].

**Figure 1:**
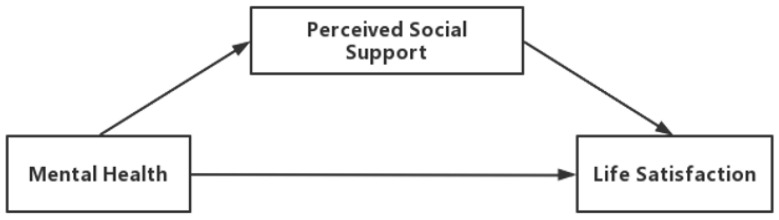
The conceptual framework of the mediating model.

Although several studies have explored the relationship between mental health and life satisfaction, few studies have clarified the mechanism underlying the relationship between mental health and life satisfaction among a large sample of the whole population. Therefore, we used a cross-sectional study to examine the relationship between mental health and life satisfaction, focusing on the mediating role of perceived social support in the relationship between mental health and life satisfaction, among the residents in Taian City, China. The conceptual framework of the mediating model is shown in Figure 1.

## 2. Materials and Methods

### 2.1. Study Design and Sample

Our data were collected from the 2020 Household Health Interview Survey in Taian City, China. This study used the stratified multistage random sampling method to conduct a questionnaire survey. Participants were selected from all six administrative districts (four counties and two districts). First, according to the level of socioeconomic development and geographical position, 3 or 4 sub-districts/towns were randomly selected from each district or county in Taian City, and a total of 20 sub-districts/towns were selected [29]. Secondly, 8 villages and 8 committees were selected separately from each town and sub-district, and a total of 160 villages/committees were selected. Finally, we randomly selected 50 households in each village/committee that made up the total sample. In total, the sample consisted of 8542 individuals in 7921 households. The response rate was 99.72%. After deleting missing data, 8500 residents aged 15 years and above were included in the study. All participants were interviewed face-to-face through paper questionnaires by trained interviewers at the participant’s home.

### 2.2. Measurement

The Mental Health Inventory (MHI) was gradually developed as the part of the National Health Insurance study [30]. Subsequently, the original 18-item scale was developed into a simplified 5-item scale. The 5-item Mental Health Inventory (MHI-5) has been widely used in quality-of-life assessment as a subscale of Mental Health in the SF-36 scale [31]. It consists of five items, each with six alternative answers assigned 1 to 6 points. Each person’s total score ranges between 5 and 30. After standardization, the total score ranges between 0 and 100 points. The higher the total score, the better the mental health status [32]. MHI-5 is even recommended to be used by a European framework [33]. The advantages of the scale are mainly reflected in its simplicity and ease of operation. The short items are helpful to improving the compliance of the respondents and the effective recovery rate of the questionnaire.

The Satisfaction with Life Scale (SWLS) [34]—The scale’s 5 items are: “my life is very perfect”, “I am satisfied with my life”, “my life is roughly in line with my ideal”, “so far, I have got the important things I want in life”, and “if I live once again, I also will not make any changes to the existing life”. Each item uses Likert’s 7-point scoring method, with 1–7 points from “strongly disagree” to “strongly agree”, and the total score was 5–35 points. The higher the score, the higher the life satisfaction.

The Perceived Social Support Scale (PSSS) is used to describe social support, including family support (4 items), friend support (4 items), and other support (4 items) [35]. Hence, there are 12 items in 3 dimensions. Each item uses the Likert 7-point scoring method, with 1–7 points scored from “very inconsistent” to “very consistent”, and the total score ranging from 12 to 84 points. The higher the total score, the higher the degree of social support people feel. The total scores of social support are 12–36, 37–60, and 61–84, for low social support, moderate social support, and high social support, respectively.

### 2.3. Control Variables

We considered several potential confounding variables in the analysis: sociodemographic characteristics included gender (male or female), age (years), educational level (primary school or below, junior middle school, senior high school, college or above), marital status (non-married or married), residence (urban or rural), employment status (non-active or active), and annual household income (CNY). We also included other variables, including chronic diseases status and urban basic medical insurance.

### 2.4. Statistical Analyses

Firstly, we described the sample’ demographic characteristics using frequencies (percentage). Secondly, Pearson correlation coefficient was used to analyze the correlation among mental health, perceived social support, and life satisfaction. Thirdly, we controlled for gender, age, educational level, marital status, residence, employment status, annual household income, chronic diseases, and urban basic medical insurance in the analysis, and then examined the mediating effect of perceived social support on mental health and life satisfaction, using SPSS 26.0 (IBM, Armonk, NY, USA). All regression coefficients were tested by the bias-corrected percentile bootstrap method. If the 95% CI did not include 0, it meant that the results of the data were significant.

## 3. Results

### 3.1. Sociodemographic Characteristics of the Participants

A total of 8500 residents aged 15 years and above were surveyed in Taian City, China. There were 5625 females (66.18%). A total of 3282 (38.61%) were aged from 40 to 59; 3936 (46.31%) had primary school education or below; 7210 (84.82%) were married; 5721 (67.31%) lived in rural areas; and 4280 (50.35%) were employed. The annual household income of 2099 participants (24.69%) was between CNY 10,000 and 30,000. There were 4293 (50.51%) residents without chronic diseases; 7407 (87.14%) were covered by urban resident basic medical insurance (URBMI) (Table 1).

### 3.2. Correlation between Key Variables

The average score of life satisfaction was 24.60 ± 4.12. The results of statistical analysis showed that marital status, residence, and employment status had statistically significant differences in the scores of residents’ life satisfaction (t = −3.052, −0.135, 1.109, *p* < 0.05). The results of statistical analysis showed that age and education level had statistically significant differences in the scores of residents’ life satisfaction (F = 57.275, 7.736, *p* < 0.05).

### 3.3. Correlation Analysis of Mental Health, Perceived Social Support and Life Satisfaction

The mean, SD, and correlation coefficients of each variable are shown in Table 2. Pearson correlation analysis showed that mental health was positively correlated with life satisfaction (r = 0.249, *p* < 0.001), mental health was positively correlated with perceived social support (r = 0.241, *p* < 0.001), and perceived social support was positively correlated with life satisfaction (r = 0.248, *p* < 0.001).

### 3.4. Mediating Effect of Perceived Social Support on Mental Health and Life Satisfaction

It can be seen from Table 3 and Table 4 that mental health had a significantly correlated effect on life satisfaction (β = 0.068, t = 23.002, *p* < 0.001). Mental health positively predicted perceived social support (β = 0.033, t = 23.752, *p* < 0.001). Furthermore, the predictive effect of mental health on life satisfaction was still significant after adding the mediating variable of perceived social support (β = 0.015, t = 23.863, *p* < 0.001). Perceived social support also positively predicted life satisfaction (β = 0.061, t = 18.483, *p* < 0.001).

In addition, the upper and lower bounds of the bootstrap 95% CIs of the direct impact of mental health on life satisfaction and the mediating effect of perceived social support on mental health and life satisfaction did not include 0 (Table 3), indicating that perceived social support had a significant mediating effect. The mediating effect value was 0.056, and the 95% CI was 0.047–0.065, accounting for 21.04% of the total effect. This suggests that perceived social support plays a partial mediating role in the relationship between mental health and life satisfaction.

## 4. Discussion

Based on previous literature, we investigated the mediating role of perceived social support on mental health and life satisfaction among the residents in Taian City, China. Our findings suggest that the impact of mental health on residents’ life satisfaction is partly mediated by perceived social support.

The results of statistical analysis showed that the differences in residents’ life satisfaction in terms of marital status, residence and employment status were statistically significant. These results are consistent with a study by Owusu Ansah et al. [36,37]. Owusu Ansah, K. analyzed cross-sectional data, including a sample of 20,059 women and men of ages ranging from 15 to 49 years [36]. In his study, marital status, wealth index, and region of residence were found to be significantly correlated with life satisfaction. Hsu, C. Y. pointed out that the level of life satisfaction decreased with the increase in neighborhood poverty rate. The negative effect on life satisfaction was more significant among those living in poverty, compared with those living in public housing [37]. Life satisfaction showed statistically significant differences in scores of age and education level; this result was consistent with the results of WANG S Q et al. [38,39,40]. Wang, S. Q. examined 1404 persons from universities, factories, companies, and elderly centers in Changchun, China. His study proved that both age and education level affect life satisfaction [39]. Blanchflower, D. G. showed that happiness depended on age in a curved relationship [38]. Melin, R. selected 1207 women and 1326 men aged 18–64 years in Sweden and confirmed education level had a predictive effect on satisfaction [40].

Similar to previous studies, there was a significant correlation among mental health, perceived social support, and life satisfaction, which was consistent with the results of WANG S Q et al. [39]. A study in China showed that key factors for life satisfaction included age, good self-rated health, trust in the community, satisfactory mental health, and communication with the neighbors [39]. Our study showed that mental health was associated with perceived social support, which was similar to previous studies [41,42,43,44]. Coyne, J. C. confirmed the effects of social support on relieving stress and its direct impact on mental health [41]. Cohen, L. H. showed that mental health promoted the formation of good social adaptability [42]. Pettit J.W. examined associative models of the relations between depressive symptoms and perceived social support from family and friends in 816 emerging adults. Results indicated that the fewer the depressive symptoms people had, the more social support they felt [43]. Węziak-Białowolska, D surveyed 41,000 residents from 79 European cities; this study revealed the importance of specific citizens and neighborhoods on urban quality of life [44]. People who are more psychologically healthy can easily obtain harmonious interpersonal relations in the communication with various people. Therefore, mental health affects social stability and development [45]. Preventing mental illness can lead to higher levels of perceived social support. Our study showed that the more psychologically healthy residents were, the higher their life satisfaction. This observation was consistent with previous findings [46,47]. Power, M. J, reported that differing health status had an impact on quality of life [46]. Paskulin LMG examined the factors contributing to quality of life of older adults in regions of Canada and Brazil. The results confirmed that health satisfaction was the largest contributor to a high quality of life [47]. Residents with good mental health have more positive emotions, and less anxiety, depression, and other negative emotions. Thus, residents with good mental health are more likely to feel happiness from others and have a higher life satisfaction.

The results of the mediating effect further indicated that mental health can directly affect residents’ life satisfaction, and indirectly affect residents’ life satisfaction through perceived social support. Our study showed a positive correlation between perceived social support and life satisfaction, which was consistent with previous studies [48,49,50,51,52]. Vázquez, C. revealed that life satisfaction was significantly correlated with social support in a representative sample of Spanish adults [48]. Ali, A. also proved that social support was one of the most important predictors of physical health and well-being for everyone, ranging from childhood to older adults [49]. Tesch-Romer, C. explored the role of the family and its contribution to quality of life in Norway, Germany, Spain, and Israel, and found that good health and quality of life depend to a large extent on good relationships within the family [50]. Ziołkowska-Weiss found that the health condition of the individual related to life satisfaction and family relationships, and adolescents’ relationship with their parents, and family life had significant effects on perceived quality of life [51]. Sparks, M. proved that social interaction and social status were significant predictors of life satisfaction [52]. Perceived social support could provide material or informational help, enhance feelings of belonging and self-worth, and improve life satisfaction. Perceived social support played an important role in shaping life satisfaction.

Although our study had a large representative sample and used a cross-sectional study to clarify the mechanism underlying the relationship between mental health and life satisfaction, it has several limitations. First, data are cross-sectional, and causal conclusions cannot be drawn. Second, perceived social support partially mediates the relationship between mental health and life satisfaction, suggesting that there might be other mediating variables in the relationship. More potential mechanisms of the relationships need to be explored, which might be the focus of further research.

Based on the results of our study, we suggest that community health managers should fully explore the role of the family and society, and adopt various means of addressing the concerns and care of residents with mental illness [49]. In addition, community volunteer groups can actively expand cultural and recreational activities, promoting the mental health of residents, guiding residents to form a positive way of thinking, shaping a positive personality, and creating a good atmosphere in the community [39]. This would encourage the pursuit of happiness, allowing individuals to feel happy in their studies, relationships, and career development.

## 5. Conclusions

This study shed light on the underlying impact of mental health on Chinese residents’ life satisfaction in Taian City, China. This relationship was found to be partly mediated by perceived social support. Based on the current situation of Chinese residents’ life satisfaction, there is an urgent need to adopt multi-layered management strategies to improve peoples’ life satisfaction. Primary health care should pay attention to the residents’ mental health, strengthen the social support for residents, and carry out intervention and treatment for residents with mental disorders, so as to improve the residents’ life satisfaction.

## Figures and Tables

**Table 1:** Demographic characteristics of residents’ life satisfaction in Taian City, China, (N = 8500).

Characteristics	Total	Proportion (%)	SWLS ($\bar{x}$ ± s)	t/F	*p*-Value
Gender				1.916 ^a^	0.216
Male	2875	33.82	24.72 ± 4.26		
Female	5625	66.18	24.54 ± 4.04		
Age				57.275 ^b^	<0.001
15~39	1347	15.85	23.51 ± 4.56		
40~59	3282	38.61	24.44 ± 4.15		
60~69	2281	26.84	24.95 ± 3.94		
>70	1590	18.71	25.35 ± 3.67		
Education level				7.736 ^b^	<0.001
Primary school or below	3936	46.31	24.78 ± 4.03		
Junior middle school	2759	32.46	24.50 ± 4.15		
Senior high school	1221	14.36	24.57 ± 4.15		
College or above	584	6.87	23.96 ± 4.38		
Marital status				−3.052 ^a^	<0.001
Non-married	1290	15.18	24.28 ± 4.47		
Married	7210	84.82	24.66 ± 4.05		
Residence				−0.135 ^a^	0.029
Urban	5721	67.31	15.80 ± 11.51		
Rural	2779	32.69	19.44 ± 12.81		
Employment status				^a^	0.039
Non-active	4220	49.65	24.65 ± 4.10		
Active	4280	50.35	24.55 ± 4.14		
Annual household income (RMB)				2.147 ^b^	0.072
≤10,000	1615	19.00	24.50 ± 4.25		
10,001~30,000	2099	24.69	24.45 ± 4.16		
30,001~50,000	1795	21.12	24.62 ± 4.07		
50,001~70,000	1155	13.59	24.84 ± 4.06		
>70,000	1836	21.60	24.69 ± 4.03		
Chronic diseases status				0.214 ^a^	0.418
No	4293	50.51	24.61 ± 4.12		
Yes	4207	49.49	24.59 ± 4.12		
Urban basic medical insurance				2.567 ^b^	0.077
Urban employee basic medical insurance (UEBMI)	993	11.68	24.88 ± 4.05		
Urban resident basic medical insurance (URBMI)	7407	87.14	24.56 ± 4.12		
Other	100	1.18	24.59 ± 4.71		

^a^, *t*-test; ^b^, ANOVA analysis.

**Table 2:** Pearson correlation coefficients of main variables among the participants in Taian City, China, 2020 (N = 8500).

Variable	M ± SD	Mental Health	Life Satisfaction	Perceived Social Support
Mental health	26.597 ± 3.719	1		
Life satisfaction	24.602 ± 4.119	0.249 **	1	
Perceived social support	68.446 ± 11.176	0.241 **	0.248 **	1

M ± SD: mean ± standard deviation. ** *p* < 0.001.

**Table 3:** The mediating effect of perceived social support on mental health and life satisfaction among the participants in Taian City, China, 2020 (N = 8500).

Predictors	Life Satisfaction		Life Satisfaction		Perceived Social Support	
	β	t	β	t	β	t
Gender	0.001	0.096	0.009	0.840	0.038	3.435 **
Age	0.216	15.703 **	0.207	14.644 **	−0.045	−3.195 *
Education level	−0.014	−1.058	−0.004	−0.269	0.046	3.519 **
Marital status	−0.033	−3.107 *	−0.040	−3.737 **	−0.035	−3.234 *
Residence	−0.017	−1.562	−0.021	−1.788	−0.014	−1.211
Employment status	−0.010	−0.919	−0.014	−1.251	−0.019	−1.627
Annual household income	0.072	6.026 **	0.085	6.934 **	0.059	4.803 **
Chronic disease	0.037	3.144 *	0.053	4.412 **	0.074	6.150 **
Urban basic medical insurance	−0.007	−0.587	−0.002	−0.135	0.024	2.017
Mental health	0.188	17.949 **	0.239	22.817 **	0.231	22.088 **
Perceived social support	0.217	20.507 **				
R^2^	0.134		0.091		0.089	
F	119.441		85.125		83.231	

* *p* < 0.01, ** *p* < 0.001.

**Table 4:** Total effect, direct effect, and mediating effect.

			Boot CI		Relative Effect Value
	Effect	Boot SE	Low	High	
Total effect	0.264	0.015	0.234	0.293	
Direct effect	0.209	0.015	0.179	0.239	78.96%
Indirect effect	0.056	0.005	0.047	0.065	21.04%

Boot CI, bootstrap CI; Boot SE, bootstrap standard error.

## Data Availability

Not applicable.
